# Disease Associations among Patients Afflicted with Both Glaucoma and Age-Related Macular Degeneration

**DOI:** 10.3390/jcm13195941

**Published:** 2024-10-06

**Authors:** Lauren Dimalanta, Kishan Pithadia, Nathan T. Shenkute, Bryan Strelow, Zhidong Zhang, Jan Ulrich, Alice Y. Zhang, David Fleischman

**Affiliations:** 1 Department of Ophthalmology, University of North Carolina, Chapel Hill, NC 27514, USA; 2 Medical College of Georgia, Augusta University, Augusta, GA 30912, USA; 3 College of Education and P-16 Integration, University of Texas-Rio Grande Valley, Brownsville, TX 78539, USA

**Keywords:** glaucoma, age-related macular degeneration (AMD), heart failure (HF), dementia

## Abstract

**Background/Objectives**: This study investigates whether there is an increased propensity to systemic conditions in patients with both age-related macular degeneration (AMD) and glaucoma in order to provide greater insight into patients’ overall health and response to physiologic stress. **Methods**: A large retrospective dataset review was conducted between April 2004 and June 2018, distinguishing four groups based on international classification of diseases (ICD) codes: glaucoma only, AMD only, glaucoma and AMD, and cataracts only (as an age-matched control). The systemic disease prevalence of each group was calculated, and a Friedman analysis was used to compare the prevalence between the groups. **Results**: This study identified 5243 patients with glaucoma only, 6726 with AMD only, 402 with combined disease, and 25,450 with cataracts only. Age and racial distributions varied between groups in a predictable manner. Two conditions, heart failure (HF) and dementia, had a statistically higher prevalence in patients with both glaucoma and AMD compared to those with glaucoma alone (HF *p* = 0.036, dementia *p* = 0.024) and cataracts alone (HF *p* = 0.003, dementia *p* = 0.036). There was no significant difference observed in terms of ethnicity and gender among the different disease groups (*p* > 0.05). **Conclusions**: Both AMD and glaucoma individually portend a higher rate of comorbidities than age-matched controls. Patients with concomitant AMD and glaucoma demonstrate a uniquely higher prevalence of heart failure and dementia than those with either disease alone. The underlying association and pathologic mechanisms warrant further investigation to improve the overall health management and prognostication for these individuals.

## 1. Introduction

The concurrence of two separate ophthalmic diseases in a single patient raises questions regarding potential underlying factors within basic physiologic systems that may predispose patients to clinical illness, such as stress maladaptation, nutrient deficiency, or inflammatory, metabolic, or cardiovascular dysfunction. Age-related macular degeneration (AMD) and glaucoma are both leading causes of vision loss, which preferentially affect elderly patients. In the United States, AMD affects nearly 11 million Americans, and glaucoma affects roughly 2.9 million [1,2]. AMD typically causes central visual loss, while glaucoma can result in peripheral visual field loss that progresses toward the central visual axis. Patients with concurrent AMD and glaucoma present with severe vision loss affecting the entire visual field. The pathophysiology of both diseases, while not completely understood, is localized to different ocular tissues. The basic pathophysiology of AMD in the eye is tied to dysfunction of the outer retina and retinal pigmented epithelium (RPE) and the breakdown of the cellular functions that are integral in the metabolic cycle of retinal photoreceptors [3]. Glaucoma, on the other hand, frequently results from the apoptotic death of retinal ganglionic cells and optic nerve axons, which are typically tied to elevated intraocular pressures (IOP) [4]. Glaucoma has classically been considered a unique form of optic neuropathy, while AMD is strictly a retinal disease. Nevertheless, each condition can result in significant, irreversible visual impairment, and both are notably more prevalent in elderly patients [5,6]. Various pathophysiologic models have been proposed for AMD, implicating different mechanisms of pathogenesis from hypoxemia and ischemia to oxidative stress and cellular inflammation to dysregulation of lipid homeostasis [1]. The underlying pathophysiology of glaucomatous optic neuropathy has traditionally been understood in terms of mechanical forces and localized ischemia of the optic nerve. However, recent literature propose more complex cellular pathways of oxidative stress and inflammation [7,8]. Additionally, genetics, environmental stress, and lifestyle factors may also play important roles in the occurrence of these conditions. In spite of the dissimilarity of these two entities clinically, there is growing evidence to suggest that similar underlying meta-mechanisms, such as inflammation and oxidative stress, may have significant implications for a patient’s health and comorbid disease [9,10,11].

Understanding the prevalence of non-ophthalmic systemic disease associated with both AMD and glaucoma may be useful in prognosticating and managing a patient’s overall health. Patients with either glaucoma or AMD are known to have increased comorbidities compared to control populations [12,13]. Currently, there is no literature examining associations in systemic disease for patients with both glaucoma and AMD. The majority of the literature related to this topic focuses on the impact of combined glaucoma and AMD on quality of life. Ugurlu and colleagues showed that patients with either primary open-angle glaucoma or nonexudative AMD with similar visual acuity had overall impairment in their quality of life [14]. Skalicky and colleagues found that patients afflicted with both glaucoma and AMD have higher vision-related activity limitations, including difficulty walking safely [15]. While quality of life considerations are important for these patients, further study of patients with combined glaucoma and AMD also has important clinical implications such as disease prognosis, appropriate therapy modalities, response to treatment, and surgical candidacy. This study seeks to fill the gap in current knowledge on the association of systemic disease in patients with both glaucoma and AMD.

## 2. Materials and Methods

The study was a retrospective chart review approved by the University of North Carolina Institutional Review Board. The i2b2 interface was used to access the Carolina Data Warehouse, which contains de-identified demographic and clinical data for all patients seen in the University of North Carolina Health System. We queried the database for patients seen between 1 April 2004 and 1 June 2018 with the international classification of diseases (ICD)-9 and ICD-10 codes for glaucoma, AMD, and cataracts specified in Table 1. The patients were divided into four study groups corresponding to their ICD codes: those with glaucoma but not AMD, those with AMD but not glaucoma, those with both glaucoma and AMD, and those with age-related cataracts but neither glaucoma nor AMD. The final group served as an age-matched control. Each of these four groups was further sub-grouped by sex, age, and race. A limited sampling chart review was conducted, as described below, to identify the most common systemic comorbidities in our patient population for further comparative analyses. Using the ICD codes listed in Table 2, the presence or absence of systemic comorbidities was analyzed in all treatment groups. The prevalence of each comorbidity was recorded for each group.

Statistical comparisons of the prevalence of comorbidities were completed between all treatment groups using the Friedman test at a significance level of *p* < 0.05. Primary outcome measures included the presence of comorbid cardiovascular, inflammatory, and neurologic diseases. Other disease associations were documented and explored as secondary outcome measures.

### Identification of Associated Comorbidities

Charts were reviewed for 50 patients from each of the four groups. The patients reviewed were randomly selected from the most recent 100 patients seen in each of the four groups of interest. Patients were de-identified, and each patient was assigned a unique identifier. Data recorded included height, weight, sex, age at last visit with ophthalmology, race, and the patient’s problem list. Diagnoses most commonly observed in the chart review were used to generate Table 2 and guide the large dataset searches in i2b2, as noted above.

## 3. Results

After applying inclusion criteria, 5243 patients with glaucoma only, 6726 with macular degeneration only, 402 with combined disease, and 25,450 with cataracts only were identified in the i2b2 database. The age, racial, and sex demographics of each study group are reported in Table 3, Table 4 and Table 5. Age distribution analysis shows that there is a significant difference between glaucoma only and glaucoma/AMD groups for ages 45–54, 55–64, 65–75, and above 85. Racial distribution between groups did not demonstrate statistically significant results between each disease group. The sex distribution of all groups was similar (*p* > 0.05).

Table 6 demonstrates the disease prevalence for comorbid conditions among each group of ophthalmologic patients with statistical comparisons between each group. There was a significantly greater prevalence of dementia and heart failure in patients with both glaucoma and AMD (glaucoma/AMD group) compared to patients with glaucoma only (HF *p* = 0.036, dementia *p* = 0.024) and cataract only groups (HF *p* = 0.003, dementia *p* = 0.036). The glaucoma/AMD group demonstrated a higher prevalence of most comorbidities when compared to the AMD only, glaucoma only, and cataract only groups.

## 4. Discussion

To our knowledge, this study is the first to analyze comorbidities in patients afflicted with both glaucoma and AMD. Among patients with both diseases simultaneously, an increase in the prevalence of comorbid heart failure and dementia was demonstrated. We suspect that suffering from these two different disease processes simultaneously is a pseudo-surrogate marker for disease susceptibility.

Current literature suggests that AMD may be associated with an increased risk of heart failure, as evidenced by recent studies [16]. Similarly, independent prospective studies have also demonstrated that patients with primary open-angle glaucoma, a subtype of glaucoma, exhibit a higher prevalence of comorbidities such as diabetes, hypertension, hyperlipidemia, and congestive heart failure [17]. Furthermore, chronic heart failure has also been associated with glaucomatous optic nerve head alterations, classically demonstrating increased cup: disc ratio and smaller rim area [18]. Additionally, both glaucoma [19,20,21] and AMD [13,22] have been variably associated with hypertension, a high-risk comorbidity for multiple cardiovascular and cerebrovascular diseases, including heart failure. Our findings also indicate that patients with both AMD and glaucoma are typically older. Therefore, given that both AMD and glaucoma are age-related conditions, the prevalence of heart failure seen among this patient population could be attributed to an increased number of age-related cardiovascular risk factors. It is also possible that the presence of both AMD and glaucoma could indicate advanced disease or underlying pathophysiology that could potentially increase the risk of heart failure. However, it is important to note that further pathophysiological investigations are required to fully understand the underlying mechanisms linking these conditions.

The association between dementia and AMD has been well documented in previous studies. Research has shown that patients with dementia are at risk for developing AMD, and conversely, AMD was also significantly associated with an increased risk of cognitive decline [23]. This could suggest a possible bidirectional relationship between these two conditions. Furthermore, there is a growing body of evidence linking glaucoma to dementia. Studies have identified similar pathophysiological mechanisms that link both conditions, including degenerative changes within ganglion cells, accumulation of neurotoxic substances such as abnormal hyperphosphorylated tau protein, and low intracranial pressure, which can lead to a high translaminar pressure gradient and optic nerve damage [24,25,26,27,28]. Additionally, prospective and population-based studies have demonstrated that glaucoma is significantly more prevalent in patients who later develop dementia [29,30]. The complex relationship between dementia and concurrence of AMD and glaucoma is not within the purview of this study; nevertheless, our results, which demonstrate a higher prevalence of dementia among individuals with both AMD and glaucoma, are concordant with previous research and imply a potential underlying mechanism connecting these conditions.

Glaucoma has previously been associated with neurologic diseases, including Alzheimer’s disease and psychiatric disorders, as well as cardiovascular disease [12,17]. Incidence of cardiovascular and renal comorbidities, including stroke, myocardial infarction, and chronic kidney disease, has been shown to correlate with the presence of AMD [13]. Previous studies have also shown that patients with both nonexudative and exudative AMD have an increased risk of heart failure [16]. While there is some overlap in associated comorbidities like cardiovascular disease, pathophysiologic mechanisms potentially tying glaucoma and AMD together have not previously been investigated. Our study is the first to demonstrate a higher prevalence of comorbid disease in patients with both glaucoma and AMD. While the disease association found in our study does not determine causality and increased risk cannot be inferred, it does suggest that patients with both glaucoma and AMD may share some underlying physiologic dysfunction which produces a broader clinical effect.

The focus of this study was to investigate potential associations of systemic diseases with concurrent AMD and glaucoma. It is important to reiterate that our findings do not determine causality or increased risk. However, we would like to explore mechanisms that may link heart failure and dementia to AMD and glaucoma and provide possible direction for future studies on this topic.

First, inflammation has been implicated in both AMD and glaucoma. As mentioned previously, the basic pathophysiology of AMD has been linked to dysfunction of the outer retina and RPE [3]. The RPE has many functions, including maintaining the blood–retinal barrier (BRB) and supporting the photoreceptors via the transportation of nutrients and disposal of waste products [31]. As the retina ages, waste products such as oxidized lipofuscin can accumulate in Bruch’s membrane and activate processes like the complement system, which can contribute to para-inflammation, a low-grade inflammatory response to noxious stimuli that helps to remove these waste products and reestablish tissue functionality [32]. However, dysregulation of para-inflammation can cause this initially beneficial process to become harmful and can occur due to activation of inflammation at the level of the RPE cells, the innate immune system in the retina (such as with the complement system and microglia), or the systemic immune system as a whole [33]. This activation can result from the aging and subsequent accumulation of waste products, genetic predisposition, and environmental stresses; however, the common endpoint is pathologic chronic inflammation [33]. Another mechanism is age-related impairment of the BRB, which allows for the passage of inflammatory molecules and an increase in oxidative stress from unstable blood flow and reperfusion injury; both of these further contribute to the chronic inflammatory response in the retina [34,35]. Both maladaptive para-inflammation and BRB impairment have also been implicated in the development and progression of glaucoma. The trigger that turns para-inflammation into damaging, chronic inflammation in glaucoma is not well understood; however, it has been suggested that altered responses to injury and maintenance of homeostasis may be inciting factors [36]. Regardless, once para-inflammation becomes dysregulated, it can cause the release of cytokines and other inflammatory factors that damage the retinal ganglion cells as well as the trabecular meshwork, the latter of which can lead to increased IOP, a feature commonly seen in primary open-angle glaucoma [36]. The BRB has also been shown to leak around the optic nerve head in patients with glaucoma, supporting the hypothesis that there may be some inflammatory damage to the retinal ganglion cells at the level of the optic nerve head from unstable blood supply to the area [34]. These are oversimplifications of complex and multifactorial conditions; however, these illustrate possible connections between AMD and glaucoma.

Next, we would like to explore the association of heart failure and dementia with glaucoma and AMD. First, we will examine potential mechanisms connecting heart failure with glaucoma and AMD, with a focus on vascular and inflammatory etiologies. Decreased cardiac output in heart failure activates neurohormonal responses (namely, the renin–angiotensin–aldosterone and the sympathetic nervous systems) to maintain adequate cardiac function; however, this also leads to decreased cerebral perfusion, including perfusion to the eye [18]. Repeated hypoperfusion followed by reperfusion to the eye can lead to inflammation from ischemia and reperfusion injury, which, as shown above, can lead to the development and progression of both glaucoma and AMD [18,34,35]. Of note, if the above mechanisms indeed play a part in relating heart failure to glaucoma and AMD, it is important to note that we did not find other cardiovascular diseases or chronic kidney disease to be significantly associated with glaucoma and AMD in our investigation. However, this discrepancy could be due to these mechanisms being specific to heart failure, the discussed mechanisms not actually having major roles, differential compensatory mechanisms, and other factors that have not yet been considered. Furthermore, as our aim was to evaluate the association of disease, we did not assess the order of diagnosis or severity of these conditions, which could also illuminate the pathophysiology that relates to these three conditions. Regarding the mechanisms connecting dementia with glaucoma and AMD, again, though there is no clear pathophysiologic pathway linking these conditions, a few have been suggested. Ashok et al. proposed that similar to Alzheimer’s disease, accumulation of AB and phosphorylated tau in drusen and retinal ganglion cells, respectively, causes inflammation and release of reactive oxygen species, leading to the death of RPE and retinal ganglion cells [37]. In a study by Maran et al., it was shown that activation of the NLRP3 inflammasome was implicated in all three of these conditions, suggesting a potential inflammatory component [35].

We will briefly touch on possible mechanisms relating to heart failure and dementia. It has been estimated that one-fifth of people with heart failure who are older than 65 years old also have dementia and that 35% to 50% of patients with heart failure will also have cognitive impairment [38,39]. One potential mechanism that relates these two diseases is decreased cerebrovascular flow. The neurohormonal responses to decreased cardiac output can lead to increased vasoconstriction in the brain and reduced cerebral blood flow, which is noted to be a prominent cause of cognitive impairment in patients with heart failure and has been correlated with the severity of dementia [18,39,40]. It has also been suggested that changes in the areas of the brain responsible for controlling the autonomic nervous system can contribute to the progression of heart failure, suggesting a bidirectional path in which heart failure and dementia contribute to each other’s evolvement [39]. While brief, it is important to acknowledge the interactions that could make it appear more likely for heart failure and dementia to occur concurrently.

Finally, the following are mechanisms that could connect heart failure, dementia, glaucoma, and AMD. First, we return to vascular etiologies. As mentioned above, heart failure can lead to decreased cerebrovascular blood flow, which has been associated with dementia and can also lead to decreased ocular perfusion. The resultant ischemia in the eye, followed by reperfusion, can cause inflammation which, either by itself or in addition to chronic ongoing inflammation, can lead to the development and progression of glaucoma and AMD. Second, we can also consider inflammatory ties. In addition to the possible inflammation from reperfusion injury, there could be systemic inflammation as well. In a study by Arvunescu et al., it was suggested that, along with recurrent acute episodes of decompensation, a low level of systemic inflammation could be responsible for the maintenance and progression of heart failure [41]. This could represent a state of increased pro-inflammatory cytokines and other inflammatory molecules circulating throughout the body which could trigger dysregulation of mechanisms that are responsible for homeostasis of the immune system in the immune-privileged tissues, like the brain and retina. Lastly, we cannot ignore age as a risk factor, as increased age can lead to the breakdown of normal functions, including those meant to control inflammation, as well as an accumulation of oxidative stress and waste products, which can serve as triggers of inflammation.

Comparative analysis of the study groups does show that the glaucoma/AMD group and AMD only group were significantly older than the glaucoma only group and cataract only group based on median age. The median age was similar in the combined group and the AMD group, suggesting that although age is a known risk factor for many of the comorbid diseases studied, the higher prevalence of heart failure and dementia in the combined group may not be driven by factors other than age. The combined AMD and glaucoma groups, however, do likely represent more systemically ill populations with a higher prevalence of other comorbidities when compared with the glaucoma only groups. It seems likely that older age is a contributor to the increased incidence of the other comorbidities studied between the combined group and single disease groups. The overall systemic illness of patients with combined glaucoma/AMD, in addition to their increased visual disability from ophthalmic disease, suggests that these patients are at high risk for morbidity and adverse events.

The racial distribution of study groups followed known epidemiological trends, with a higher prevalence of white patients in the AMD only group and a higher prevalence of black patients in the glaucoma only group. The combined AMD/glaucoma group showed similar racial distributions to the cataract only control group suggesting that race may not be a significant contributor to the prevalence of comorbid conditions in our study.

The study has several limitations. The time frame of interest spanned from 2004 to 2018, which included the 2015 transition to the ICD-10 classification system. As a result, there may be some inconsistency between the legacy classification in ICD-9 and new ICD-10 codes caused by changes in the descriptive diagnoses for ICD-10. The study was also limited to only primary glaucoma types to prevent confounding from systemic diseases that may cause secondary glaucoma (such as carotid disease, diabetes, or neoplasm). Furthermore, as only aggregate data were analyzed, our study did not account for the course, severity, or causes of ophthalmologic conditions and comorbidities. In addition, the discrepancies between the total for race, age, and sex versus overall totals are attributable to the lack of demographic data on some charts. Finally, given the retrospective nature of our sampling, causality between ophthalmologic conditions and comorbidities cannot be demonstrated.

The goal of this study was to broadly determine disease associations in individuals with both glaucoma and AMD. Despite its limitations, our study does analyze a large cohort of ophthalmology patients and provides evidence of unique prevalence in comorbid conditions for patients with both glaucoma and AMD. Additionally, it corroborates literature that suggests AMD and glaucoma are both individually associated with heart failure and dementia. These associations merit further investigation and validation on other datasets. Further studies may investigate the time course and progression of comorbidities, specifically HF and dementia, as well as of ocular conditions in patients with both glaucoma and AMD to further elucidate the specific pathologic mechanisms that may be driving these associations.

## Figures and Tables

**Table 1:** ICD-9 and ICD-10 ophthalmic diagnosis codes used to generate the study population.

Disease	ICD Codes
Glaucoma	365.1: Open-angle glaucoma 365.2: Primary angle-closure glaucoma H40.1: Open-angle glaucoma H40.2: Primary angle-closure glaucoma
Macular Degeneration	362.50: Macular degeneration (senile), unspecified 362.51: Nonexudative senile macular degeneration 362.52: Exudative senile macular degeneration H35.30: Unspecified macular degeneration H35.311: Nonexudative age-related macular degeneration, right eye H35.312: Nonexudative age-related macular degeneration, left eye H35.313: Nonexudative age-related macular degeneration, bilateral H35.31: Nonexudative age-related macular degeneration H35.32: Exudative age-related macular degeneration
Cataracts (control)	366.1: Senile cataract H25: Age-related cataract

**Table 2:** ICD-9 and ICD-10 diagnosis codes derived from chart review for systemic comorbidities.

Diagnosis	ICD Codes
Angina Pectoris	I20, 413
Chronic Kidney Disease	585, N18
Coronary Artery Disease	I25, 414
Dementia	F01, F02, F03, G30, 294.1, 294.2, 290, 331.0
Diabetes Mellitus	250, 249, E08-E13
Heart Failure	428, I50
Hyperlipidemia	E78.2, E78.4, E78.5, E78.00, 272.0, 272.2, 272.4
Hypertension	I1, 401
Lupus	M32
Multiple Sclerosis	G35
Myocardial Infarction	I21, 410
Neoplasm, Benign	D21
Neoplasm, Malignant	C00-C96
Obesity	E66.0, E66.1, E66.2, E66.8, E66.9, 278.00, 278.01, 278.03
Sarcoidosis	D86
Stroke	I63.3, I63.5, I63.6, I63.8, I63.9, 434.0, 434.9

**Table 3:** Age breakdown of study groups.

Age Range	Glaucoma, *n* (%)	AMD, *n* (%)	Both, *n* (%)	Cataracts, *n* (%)
18–34	76 (1)	15 (0)	0 (0)	104 (0)
35–44	125 (2)	26 (0)	0 (0)	244 (1)
45–54	277 (5)	60 (1)	0 (0)	954 (4)
55–64	757 (14)	206 (3)	0 (0)	3686 (14)
65–74	1427 (27)	853 (13)	41 (11)	8921 (35)
75–84	1432 (27)	1712 (26)	95 (24)	8123 (32)
>=85	1135 (22)	3840 (57)	253 (65)	3391 (13)
Total	*n* = 5229	*n* = 6712	*n* = 389	*n* = 25,423

AMD = Age-related Macular Degeneration.

**Table 4:** Race breakdown of study groups.

Race	Glaucoma, *n* (%)	AMD, *n* (%)	Both, *n* (%)	Cataracts, *n* (%)
Native American	15 (0.3)	0 (0)	0 (0)	67 (0.3)
Asian	129 (2)	67 (1)	0 (0)	503 (2)
Black	1962 (38)	256 (4)	44 (11)	4868 (19)
Hawaiian	0 (0)	0 (0)	0 (0)	12 (0.05)
Other	336 (6)	208 (3)	17 (4)	1521 (6)
Refused	0 (0)	0 (0)	0 (0)	14 (0.1)
Unknown	183 (3)	234 (3)	0 (0)	711 (3)
White	2607 (50)	5930 (89)	328 (84)	17,727 (70)
Total	*n* = 5232	*n* = 6695	*n* = 389	*n* = 25,423

AMD = Age-related Macular Degeneration.

**Table 5:** Sex breakdown of study groups.

Gender	Glaucoma, *n* (%)	AMD, *n* (%)	Both, *n* (%)	Cataracts, *n* (%)
Female	2900 (55)	4310 (64)	248 (62)	14,805 (58)
Male	2336 (45)	2401 (36)	153 (38)	10,616 (42)
Total	*n* = 5236	*n* = 6711	*n* = 401	*n* = 25,421

AMD = Age-related Macular Degeneration.

**Table 6:** Risk factors and comorbidities and their association with glaucoma and AMD.

Prevalence of Systemic Disease and Events among Ophthalmologic Patients						
Systemic Disease	% Glaucoma and AMD	% Glaucoma	*p*-Value ^+^	% AMD	% Cataract	*p*-Value ^++^
Angina Pectoris	7.0	6.7	1.000	5.9	6.1	0.782
Coronary Artery Disease	32.8	24.9	0.294	34.9	27.1	0.439
Chronic Kidney Disease	25.4	20.5	0.555	23.5	19.4	0.366
Myocardial Infarction	10.7	6.4	0.225	9.4	6.7	0.346
Dementia	23.1	9.5	0.024 *	18.3	6.6	0.003 *
Heart Failure	33.3	18.1	0.036 *	28.1	17.5	0.036 *
Obesity	14.7	22.1	0.249	13.7	23.5	0.149
Diabetes	29.6	37.3	0.392	28.4	38.5	0.278
Hypertension	78.1	69.0	0.458	74.7	70.0	0.511
Hypercholesterolemia	60.4	57.4	0.782	59.4	58.6	0.927
Stroke	8.0	7.4	0.796	9.7	7.3	0.796
Malignant Neoplasm	26.9	20.3	0.307	26.9	22.2	0.475
Benign Neoplasm	36.6	34.3	0.722	24.7	33.5	0.722

^+^ Comparison of glaucoma patients to glaucoma/AMD patients. ^++^ Comparison of AMD patients to glaucoma/AMD patients. * Indicates significance of *p* < 0.05. AMD = Age-related Macular Degeneration.

## Data Availability

Data are available upon request.
